# Downregulation of CD73 associates with T cell exhaustion in AML patients

**DOI:** 10.1186/s13045-019-0728-3

**Published:** 2019-04-24

**Authors:** Yaxian Kong, Bei Jia, Chenchen Zhao, David F. Claxton, Arati Sharma, Charyguly Annageldiyev, Joseph S. Fotos, Hui Zeng, Robert F. Paulson, K. Sandeep Prabhu, Hong Zheng

**Affiliations:** 1 Penn State Hershey Cancer Institute, Penn State University College of Medicine, Hershey, PA 17033 USA; 2 Beijing Key Laboratory of Emerging Infectious Diseases, Institute of Infectious Diseases, Beijing Ditan Hospital, Capital Medical University, Beijing, 100015 China; 3 Department of Intensive Care Unit, Affiliated Hospital of Yangzhou University, Yangzhou University, Yangzhou, 225000 Jiangsu China; 4 Department of Pharmacology, Penn State University College of Medicine, Hershey, PA 17033 USA; 5 Department of Radiology, Penn State University College of Medicine, Hershey, PA 17033 USA; 6 Department of Veterinary and Biomedical Sciences, Penn State University College of Agricultural Sciences, University Park, Harrisburg, PA 16802 USA

**Keywords:** CD73, AML, T cell exhaustion, PD-1, TIGIT

## Abstract

**Background:**

Successful treatment for acute myeloid leukemia (AML) remains challenging. Inhibiting immune checkpoint to enhance anti-tumor response is an attractive strategy for effective leukemia therapeutics. CD73 is a recently recognized immune checkpoint mediator that is highly expressed on tumor cells and stromal cells in tumor microenvironment. The ectonucleotidase activity of CD73 catalyzes AMP to adenosine, which subsequently inhibits anti-tumor immune responses. In this study, we aim to explore the effect of CD73 in AML.

**Methods:**

Peripheral blood samples collected from patients with newly diagnosed AML (*n* = 27) were used in this study. CD73 expression on each immune cell component was examined by flow cytometry. Phenotypic study of CD73-expressing T cells and analysis of the correlation between CD73 and other immune checkpoints were performed using flow cytometry-based assays. Functional status of CD73^+^ vs. CD73^−^ T cells was assessed in an in vitro cytokine release assay upon CD3/CD28 antibody stimulation.

**Results:**

In contrast to the long recognized immune suppressive effect of CD73-adenosine signaling in tumor tissue, we made a striking observation that in AML, CD73 expression on CD8 T cells associates with an increased immune response. CD73^+^ CD8 T cells are more functional, whereas CD73^−^ CD8 T cells exhibit features of exhaustion manifested by high expression of inhibitory receptors such as PD-1 and TIGIT, increased intracellular expression of Eomes, reduced capacity of cytokine production, and high susceptibility to apoptosis.

**Conclusions:**

Our data highlight the potential of CD73 as a double-edged sword in anti-leukemia immunity and argue strongly for the combinational treatment by adding immune checkpoint inhibitors to the CD73-targeting approaches.

## Introduction

Recent FDA approval of eight novel agents in the last 2 years has significantly expanded options for treatment of acute myeloid leukemia (AML) [1–7]. A variety of additional leukemia therapeutics targeting specific genetic mutations or cellular processes are emerging [8–14]. Despite these exciting advances, successful treatment of AML remains challenging with 5-year survival of only 27.4% according to the National Cancer Institute (NCI). Novel effective and better tolerated leukemia therapeutics is an urgent unmet need.

Immunotherapy is promising in cancer treatment. Several immune checkpoint inhibitors, such as antibodies against programmed cell death protein 1 (PD-1) or PD-ligand 1 (PD-L1), have been FDA approved for treating multiple solid tumors and Hodgkin lymphoma [15–20]. Multiple studies including ours have demonstrated the involvement of PD-1 and other immune inhibitory pathways in the pathogenesis of leukemia progression [21–31]. Clinical translation of strategies targeting PD-1 in AML treatment is currently under active investigations [32, 33]. In fact, Daver et al. have recently reported a promising result from an early clinical trial treating AML patients with a combination of azacitidine and nivolumab, the first approved PD-1-targeting agent [34]. AML is highly heterogeneous. Identification of additional immune regulatory pathways and understanding their interactions with other pathogenesis mechanisms are crucial to develop effective immunotherapy for AML.

CD73 is an ectonucleotidase that is highly expressed on tumor cells and multiple cell components in tumor microenvironment such as stromal cells, endothelial cells, and regulatory T cells (Treg) [35]. The 5′-nucleotidase activity of CD73 on these cells catalyzes AMP to adenosine, a potent suppressor for T cell function [36, 37]. Enrichment of adenosine in tumor environment subsequently inhibits anti-tumor T cell responses [38–42]. This forms the base for targeting CD73 as optimal immunotherapy of cancer [43–45]. Blockade antibodies against CD73 are currently under active clinical studies for treating solid tumors (NCT03454451, NCT02503774). However, little is known for the effect of CD73 in AML. In order to address this important question, we performed comprehensive phenotypic and functional studies on the CD73-expressing T cells derived from peripheral blood of a cohort of AML patients (*n* = 27) at initial diagnosis.

## Materials and methods

### Patients

Peripheral blood samples of AML patients were from the tissue bank maintained by the Penn State Cancer Institute of Penn State University College of Medicine, Hershey, PA. The study was approved by the Institutional Review Board of Penn State University College of Medicine. Full written informed consent was obtained from all patients. Samples from 27 patients diagnosed with AML per WHO classification were used in the study. The average age of patients was 63 years old (range 40–80), and there were 11 males and 16 females. Risk stratification based on cytogenetics was per ELN 2017 [46]. The majority of patients carried intermediate (13 patients) or high risk (10 patients), and only 2 patients were categorized with favorable risk. Samples of 16 healthy volunteers (6 males and 10 females, age 57 ± 8 years, range, 44–70 years) were obtained as controls.

### Immunofluorescence staining and flow cytometric analysis

For surface staining, peripheral blood mononuclear cells (PBMCs) were incubated with directly conjugated mAbs for 30 min at 4 °C and then washed before analysis. Antibodies used were anti-human CD3-BV786 or CD3-APC, CD4-BV711, CD8-APC-H7, CD45RA-AF700, CD95-FITC, CD25 BV510, CD73 PE-CY7, CD11b AF700, CD45 BV786, CD14 BV711, CD56-PE-CF594, CD19-FITC, HLA-DR-PE, CD160-AF488 (BD Biosciences, San Diego, CA, USA), CCR7-BV421, PD-1-PE, 2B4-APC (BioLegend, San Diego, CA, USA), TIGIT-APC (Ebioscience, San Diego, CA, USA), LAG-3-AF700 (R&D Systems, Minneapolis, MN, USA) antibodies, and corresponding isotype controls. Data acquisition was performed on a LSR Fortessa flow cytometer (BD Biosciences), and data analysis was performed using FlowJo Software (Tree Star, Ashland, OR, USA).

### In vitro stimulation and intracellular staining

PBMCs were cultured in RPMI-1640 medium (GIBCO, Grand Island, NY, USA) containing 10% FBS and stimulated with anti-CD3/CD28 (2 μg/mL and 5 μg/mL, Ebioscience), plus Golgiplug (BD Biosciences) for 5 h. The cells were then surface stained with CD3-BV786, CD4-BV711, CD8-APC-H7, CD45RA-AF700, and CCR7-FITC and intracellularly stained with IFN-γ-PE, TNF-α-BV421, or IL-2-APC (BD Biosciences) antibodies. For EOMES staining, PBMCs were intracellularly stained with EOMES-eF610 (BD Biosciences). A Fixable Viability Dye eFluor® 506 (Ebioscience) was used to assess cell viability.

### In vitro expansion and analysis of leukemia-reactive CD8 T cells

The experiment protocol was described in our previous published studies [47]. Briefly, purified CD8 T cells derived from PBMCs of AML patients were co-cultured with T2 cells that were pulsed with 10 μM WT1_126–134_ peptide, in the presence of 50 IU IL-2 (R&D system) for 6 days. IL-2 was re-added on day 3. On day 6, CD8 T cells were re-stimulated with the peptides, plus GolgiPlug, for 5 h and followed by intracellular cytokine staining and flow cytometric analysis.

### siRNA transfection

SMARTpool Accell CD73 siRNA, Accell Non-targeting Pool, and Accell siRNA delivery media were purchased from GE Dharmacon (Lafayette, CO, USA). Control and specific siRNAs were transfected in a 96-well tissue culture plate at a final concentration of 2 μM with Accell siRNA delivery media for 72 h. Cells were then stimulated with anti-CD3/CD28 antibodies for 5.5 h for cytokine functional assay, measured by flow cytometry.

### Apoptosis analysis

PE Annexin V Apoptosis Detection Kit (BD Biosciences) was used for apoptosis assay following the manufacturer’s instructions, in combination with markers for T cells.

### Statistical analysis

Data are expressed as the mean ± standard deviation (SD). GraphPad 5 (GraphPad Software, La Jolla, CA, USA) or SPSS (IBM Corporation, New York, NY, USA) were used for statistical calculations. The normality of each variable was evaluated using the Kolmogorov-Smirnov test. In cases of normally distributed data, the comparison of variables was performed using unpaired or paired (where specified) two-tailed Student’s *t* tests for unpaired and paired data, respectively. When the data were not normally distributed, the comparison of variables was performed with a Mann-Whitney *U* test or a Wilcoxon matched-pairs signed rank test for unpaired and paired data, respectively. Comparisons of patient characteristics were analyzed using Fisher’s exact test (categorical variables) or Wilcoxon rank sum test (continuous variables). To evaluate correlation, Pearson’s correlation coefficients were used. For all analyses, *P* values less than 0.05 were considered statistically significant.

## Results

### CD73 is highly expressed on CD8 T cells in peripheral blood from AML patients

**Fig. 1:**
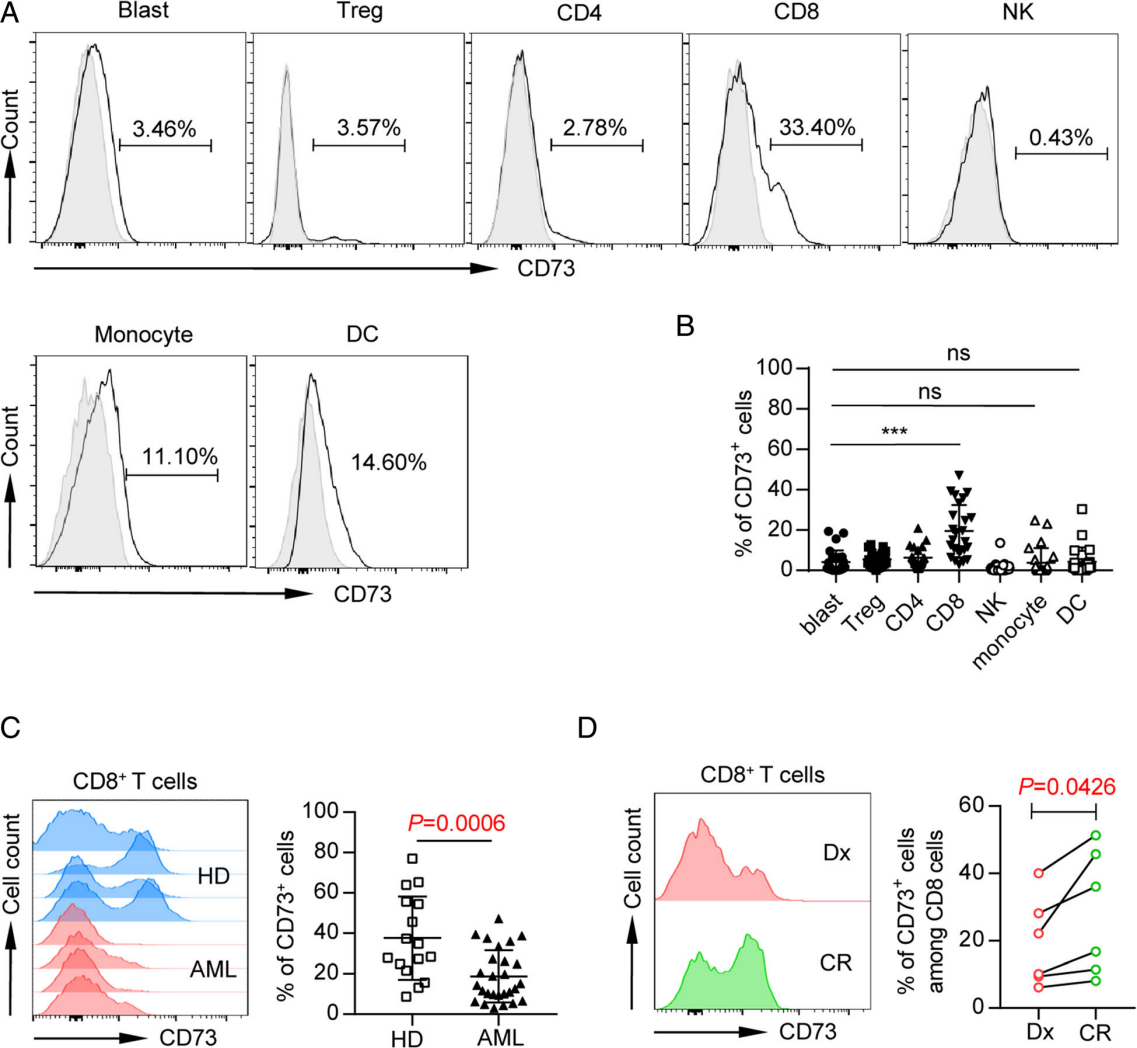
CD73 is downregulated on CD8 T cells in patients with newly diagnosed AML compared with healthy controls and patients with complete remission (CR). Flow cytometry analysis of CD73 expression was performed on PBMCs collected from AML patients at the initial diagnosis (*n* = 27), healthy donors (*n* = 16), and CR patients (*n* = 6). **a**, **b** The expression of CD73 on leukemic blasts, T cells (CD4, CD8 T cells, and Treg), natural killer cells, and myeloid cells (monocytes and dendritic cells) was assessed by flow cytometry. Representative flow data (**a**) and summary plot (**b**) of CD73 expression on indicated subsets were shown; each dot indicates one patient. *P* values were obtained by the unpaired *t* test or Mann-Whitney test. ***P < 0.001. **c** Representative histogram (left) and summary plot (right) of CD73 expression on CD8 T cells from healthy donors vs. AML patients. *P* values were obtained by unpaired *t* test. **d** Representative flow data (left) and plots (right) showing expression of CD73 on CD8 T cells from AML patients at initial diagnosis and complete remission. *P* values were obtained by paired *t* test

To determine which cell components express CD73 in a patient with AML, peripheral blood mononuclear cells (PBMCs) were assessed for CD73 expression by flow cytometry gated on markers for blast (CD45^int^, low SSC), Treg (CD45^hi^CD4^+^CD25^+^), CD4, CD8, NK (CD45^hi^CD3^−^CD56^+^), monocytes (CD45^hi^CD11b^+^CD14^hi/low^), and dendritic cells (DCs; CD45^hi^CD3^−^CD19^−^CD56^−^CD14^−^HLA-DR^+^). In contrast to the findings in previous studies of solid tumors that primary tumor or tumor cell lines highly express CD73, we observed minimal CD73 expression on AML blasts (mean frequency 4.75 ± 6.21, Fig. 1a, b), whereas the majority of patients express significant level of CD73 on their CD8 T cells (mean frequency 22.26 ± 13.79, Fig. 1a, b). There was moderate CD73 expression on monocytes and DCs, while low expression was detected on Treg, CD4 T cells, and NK cells (Fig. 1a, b). This data suggests the involvement of CD73 in CD8 T cell response in AML.

### Low CD73 expression on CD8 T cells associates with high leukemia burden

We then focused our study on CD8 T cells and compared the expression of CD73 in AML (*n* = 27) vs. healthy controls (*n* = 16). Strikingly, CD8 T cells from newly diagnosed AML patients expressed significantly lower frequency of CD73 compared with those from healthy controls (*P* = 0.0006, Fig. 1c). We further assessed the longitudinal samples collected from the same patients at the initial diagnosis and at the time of complete remission (morphologically leukemia free) post treatment. We observed a significant increase of CD73 expression on CD8 T cells upon achieving complete remission (*P* = 0.0426, Fig. 1d). Therefore, low CD73 expression on CD8 T cells associates with high leukemia burden.

### CD73 is preferentially expressed on naïve T cells

**Fig. 2:**
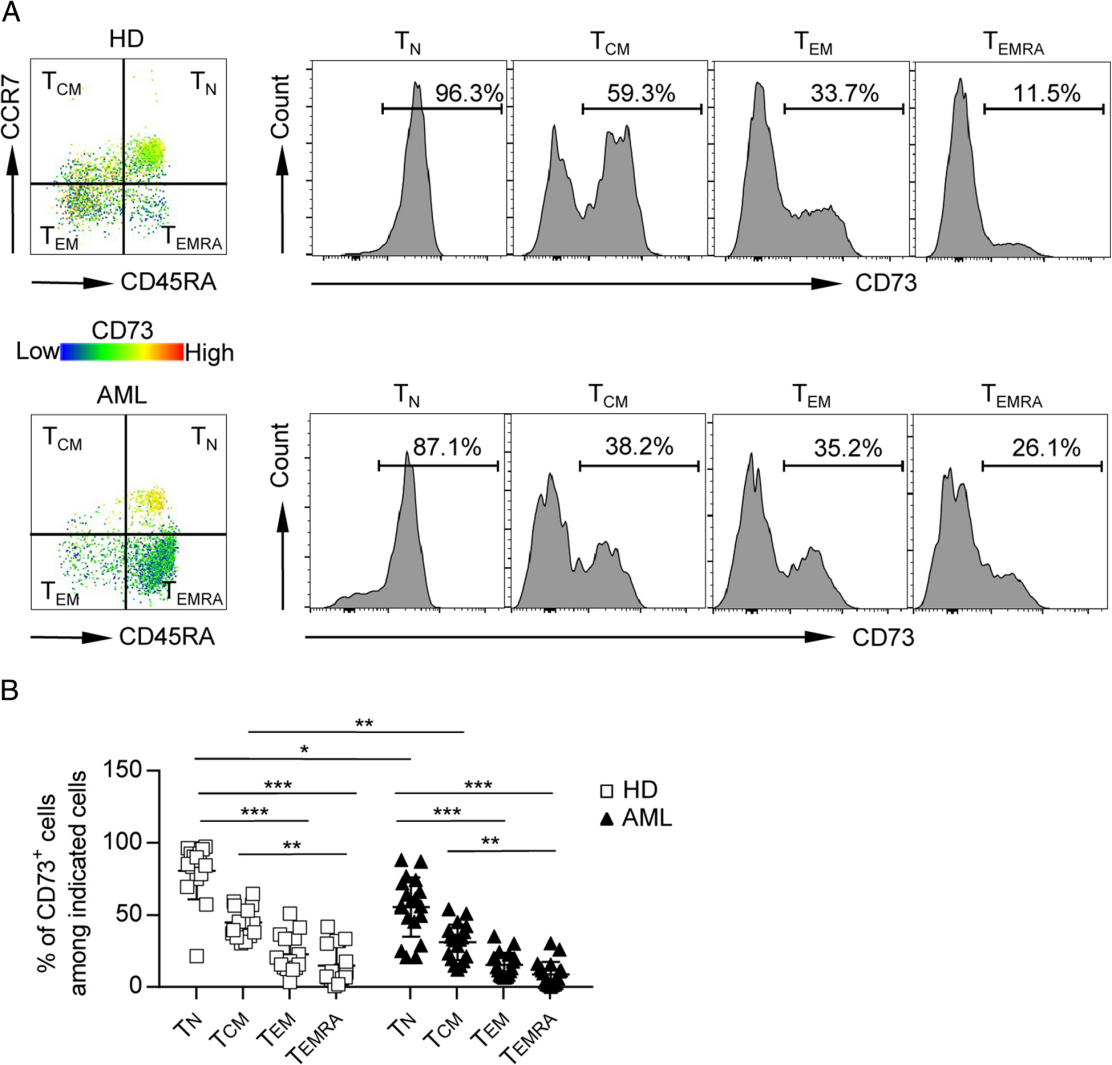
CD73 is preferentially expressed on T_N_ cells. Expression of CD73 among each subset (T_N_, T_CM_, T_EM_, and T_EMRA_) of CD8 T cells was analyzed. Representative flow data (**a**) and plots (**b**) of percentage of CD73 expression among each subset of CD8 T cells from healthy donors (*n* = 16) or AML patients (*n* = 27) are shown. *P* values were obtained by Kruskal-Wallis test followed by Dunn’s multiple comparisons test. *P < 0.05, **P < 0.01, ***P < 0.001

We next investigated the phenotypic features of CD8 T cells expressing CD73. Based on the expression of CD45RA and CCR7, T cells can be divided into four subsets: naïve T cells (T_N_; CCR7^+^CD45RA^+^), central memory T cells (T_CM_; CCR7^+^CD45RA^−^), effector memory T cells (T_EM_; CCR7^−^CD45RA^−^), and terminally differentiated effector cells (T_EMRA_; CCR7^−^CD45RA^+^). We assessed the expression of CD73 on each subset and observed significantly higher expression of CD73 on T_N_, compared to that of T_CM_, T_EM_, and T_EMRA_, indicating the downregulation of CD73 in antigen-experienced CD8 cells. This occurred in both AML patients as well as healthy controls. Although there was no difference of CD73 expression on CD8 T_EM_ and T_EMRA_ cells among healthy controls and AML patients, CD8 T_N_ and T_CM_ subset from AML patients expressed lower levels of CD73 compared with those in their counterparts from healthy controls (Fig. 2). Of note, CD73 was expressed lowest on T_EMRA_, which are considered terminal effector cells with limited functional capacity and high susceptibility to apoptosis, thus more consistent with an exhaustion phenotype. Our data demonstrated that CD73 expression is high on T_N_, but low on T_EMRA_ CD8 T cells in AML patients, indicating a phenotypical correlation between downregulation of CD73 and T cell exhaustion.

### CD73^−^ CD8 T cells from AML patients exhibited a phenotype of exhaustion and over-activation

**Fig. 3:**
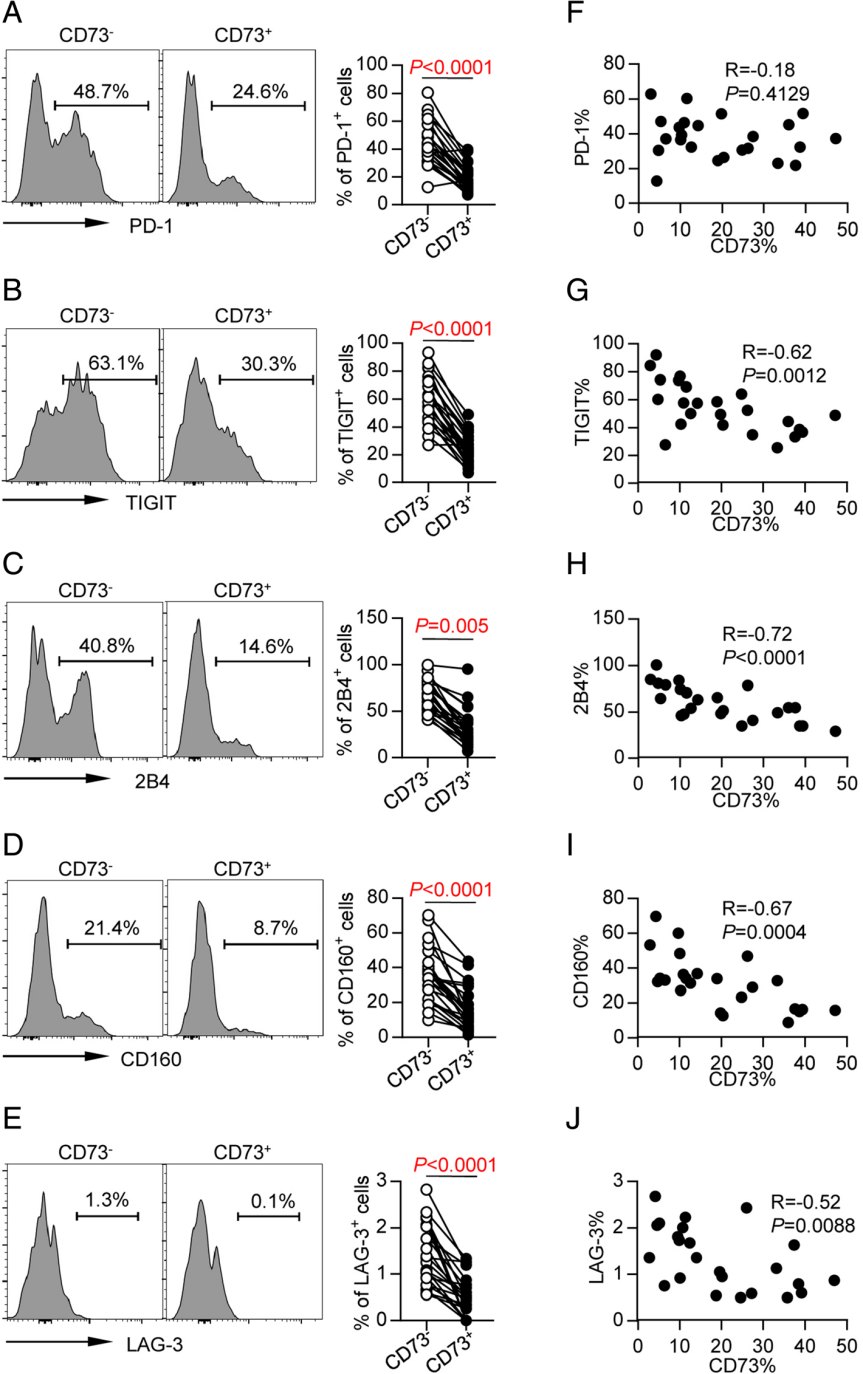
CD73 expression associates with downregulation of multiple inhibitory receptors. Flow cytometry analysis of expression of PD-1, 2B4, CD160, and LAG3 on CD73^−^ vs. CD73^+^ CD8^+^ T cells from AML patients (*n* = 25). **a**–**e** Representative flow data (left) and summary data (right) are shown. *P* values were obtained by paired *t* test (PD-1, TIGIT, LAG-3) or Wilcoxon matched-pairs signed rank test (2B4, CD160). **f**–**j** Correlative analysis of CD73 and expression of above receptors are shown. Pearson’s test was used to test for correlations

To further determine whether lower expression of CD73 on CD8 T cells from AML patients was associated with status of exhaustion, we compared the expression of a number of inhibitory receptors on the CD73^+^ vs. CD73^−^ CD8 T cells in PBMCs collected from AML patients. We observed significantly higher expression of PD-1, TIGIT, 2B4, CD160, and LAG-3 on CD73^−^ CD8 T cells, compared with that on CD73^+^ CD8 T cells (Fig. 3a–e). Additionally, expression of CD73 was inversely correlated with expression of TIGIT (*r* = − 0.62, *P* = 0.0012, Fig. 3g), 2B4 (*r* = − 0.72, *P* < 0.0001, Fig. 3h), CD160 (*r* = − 0.67, *P* = 0.0004, Fig. 3i), and LAG-3 (*r* = − 0.52, *P* = 0.0088, Fig. 3j). We did not find any direct correlation between expression of CD73 and PD-1 (*r* = − 0.18, *P* = 0.4129, Fig. 3f).

**Fig. 4:**
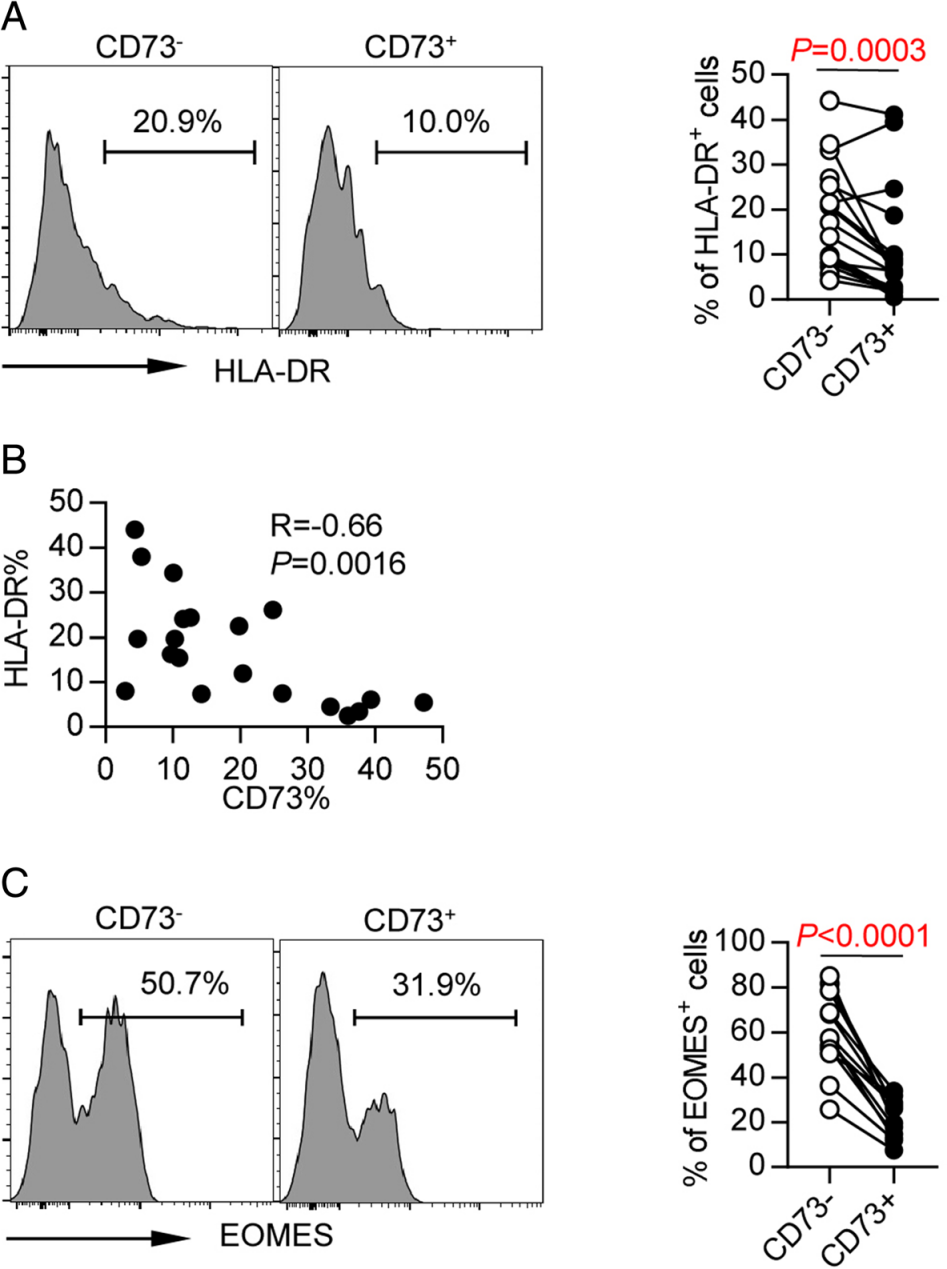
CD73^−^ CD8^+^ T cells from AML patients showed phenotypic and transcriptional defects that a consistent with exhaustion. **a** HLA-DR expression on CD73^−^ and CD73^+^ CD8 T cells from AML patients (*n* = 19). *P* values were obtained by Wilcoxon matched-pairs signed rank test. **b** Correlative analysis of CD73 and HLA-DR is shown. Pearson’s test was used to test for correlations. **c** Intracellular expression of EOMES on CD73^−^ and CD73^+^ CD8^+^ T cells from AML patients (*n* = 13). *P* values were obtained by paired *t* test

Since T cell exhaustion is a consequence of over-activation of T cells caused by high antigenic stimulation, we also analyzed the activation status of CD73^−^ CD8 T cells by measuring HLA-DR expression. We observed a significantly higher expression of HLA-DR in CD73^−^ CD8 T cells compared with that in CD73^+^ CD8 T cells (Fig. 4a). Consistently, the expression of CD73 was inversely correlated with that of HLA-DR (*r* = − 0.66, *P* = 0.0016, Fig. 4b).

Furthermore, we examined the expression of Eomesodermin (Eomes), a key transcription factor governing CD8^+^ T cell exhaustion. It has been demonstrated that Eomes^hi^ CD8 T cells are terminally exhausted and not able to be reinvigorated by PD-1 blockade. Intracellular Eomes was assessed on PBMCs from patients with newly diagnosed AML. We observed significantly higher expression of Eomes in CD73^−^ CD8 T cells than in CD73^+^ cells (*P* < 0.0001, Fig. 4c), suggesting that CD73^−^ CD8 T cells in AML are more terminally exhausted.

Taken together, our findings demonstrate that in AML patients, CD73^−^ CD8 T cells expressed high level of co-inhibitory receptors and Eomes. In addition, these cells remained in an over-activated status, therefore are more consistent with an exhausted phenotype.

### CD73^−^ CD8 T cells from AML patients are functionally deficient

**Fig. 5:**
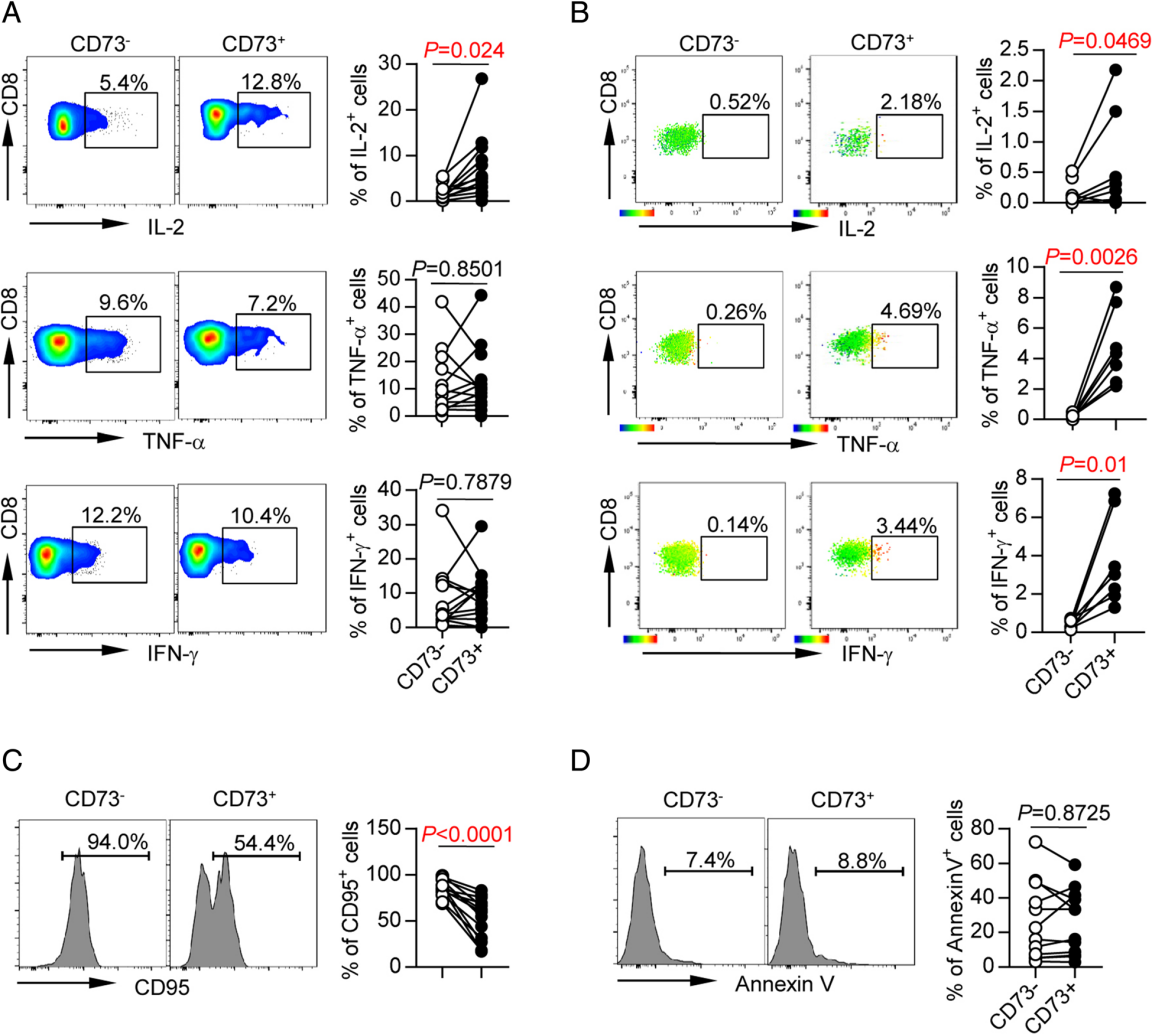
CD73^−^ CD8^+^ T cells from AML patients showed functional defects in cytokine production and high susceptibility to apoptosis. **a** Intracellular cytokine production of IL-2, TNF-α, and IFN-γ by CD73^−^ and CD73^+^ CD8 T cells from AML patients (*n* = 13) upon in vitro anti-CD3/anti-CD28 stimulation. *P* values were obtained by paired *t* test (IL-2, IFN-γ) or Wilcoxon matched-pairs signed rank test (TNF-α). **b** CD8 T cells were purified from PBMC of HLA-A*0201 AML patients at initial diagnosis. Then, they were co-cultured with T2 cells (used as antigen-presenting cells) that were pulsed with HLA-A*0201-binding WT-1_126-134_ peptide for 6 days. Then, cells were collected and intracellular staining was performed. Shown are the expression of IL-2, TNF-α, and IFN-γ in CD73^−^ vs. CD73^+^ leukemia-reactive CD8 T cells assessed by flow cytometry. In the left are representative flow cytometry data. In the right are the statistical summary plots; each dot indicates one patient. *P* values were obtained by paired Student’s *t* test or Wilcoxon signed-rank test. **c**, **d** Expression of CD95 (**c**) and Annexin V (**d**) on CD73^−^ and CD73^+^ CD8 T cells from AML patients (*n* = 13). *P* values were obtained by paired *t* test

To determine the contribution of CD73 expression to CD8 T cells’ function, we performed an in vitro assay to evaluate intracellular cytokine productions by CD8 T cells upon anti-CD3 and anti-CD28 stimulation. In order to rule out potential confounding effect of naïve T cells, which produce minimal cytokines, we specifically analyzed cytokines produced by antigen-experienced cells (T_CM_, T_EM_, and T_EMRA_). We observed a significantly lower release of IL-2 by CD73^−^ cells compared with that by CD73^+^ cells (*P* = 0.024, Fig. 5a). There were no significant differences detected between CD73^+^ and CD73^−^ cells in producing TNF-α and IFN-γ likely due to limited sample size (Fig. 5a). To further dissect the function of leukemia-reactive CD73^−^ CD8 T cells, we examined CD8 T cells for cytokine release in response to WT-1, which is a well-known tumor-associated antigen. CD8 T cells purified from PBMCs of HLA-A*0201 AML patients were co-cultured in vitro with T2 cells (used as antigen-presenting cells) that were pulsed with HLA-A*0201-binding WT-_1126-134_ peptide. We observed significantly lower production of IL-2, TNF-α, and IFN-γ by CD73^−^ CD8 T cells compared to that by CD73^+^ CD8 T cells (Fig. 5b). This important data demonstrate a less anti-leukemia T cell response in CD73^−^ CD8 T cells.

Susceptibility to apoptosis is also a hallmark for functional status of T cells. CD73^−^ CD8 T cells from AML patients showed a trend of higher susceptibility to apoptosis manifested by significantly higher expression of CD95 expression (*P* < 0.0001, Fig. 5c). Interestingly, expression of Annexin V was comparable between CD73^−^ and CD73^+^ CD8 T cells (*P* = 0.8725, Fig. 5d).

Collectively, our findings demonstrate that in AML patients, CD73^−^ CD8 T cells expressed high level of immunosuppressive molecules and were less functional, therefore consistent with exhaustion.

## Discussion

Despite the well-known immune suppressive role of the CD73 expression on tumor and stromal cells in tumor microenvironment, literature describing the direct effect of CD73 on T cells is limited. To our knowledge, this is the first study to uncover the important role of CD73 expression on CD8 T cells in AML. In contrast to the long recognized negative immune regulatory effect of CD73-adenosine signaling in tumor tissue, we made a striking observation that CD73 expression on CD8 T cells associates with an increased immune response. CD73^+^ CD8 T cells are more functional, and high frequency of this subpopulation associates with low disease burden in AML, whereas CD73^−^ CD8 T cells exhibit features of exhaustion manifested by high expression of inhibitory receptors such as PD-1 and TIGIT, increased intracellular expression of Eomes, and reduced capacity of cytokine production and high susceptibility to apoptosis. Of note, we made an effort to study the effect of knockdown CD73 on CD8 T cells from AML patients by specific siRNA. CD73 was decreased upon siRNA knockdown; however, it did not significantly alter the cytokine production by CD8 T cells (data not shown). Therefore, CD73 may regulate T cell function via an indirect mechanism. Further studies involving multiple immune components in an optimal culture system mimicking tumor microenvironment are warranted to fully address whether the correlation of CD73 to T cell function is causative. Nevertheless, our data highlight the potential of CD73 as a double-edged sword in anti-leukemia immunity. It provides a rational explanation for the controversial reports in the studies of predictive values of CD73 in clinical outcome for cancer patients. The majority of studies in cancer patients demonstrated an association of elevated CD73 to poor prognosis [48–52]. In contrast, opposite results were reported in other studies in which a correlation of high CD73 expression to a good clinical outcome was observed [53–57]. It is possible that one study mainly detected the CD73 expression on tumor cells, stromal cells, and Treg, while CD73 expression on CD8 T cells was predominantly assessed in the other study. Therefore, understanding the specific distribution pattern of CD73 in each cancer type or disease status is essential for optimal design of clinical studies targeting CD73 for cancer treatment.

We observed a significantly higher expression of CD73 on naïve T cells. This is in line with the previous report that CD73 suppress T cell differentiation via autocrine adenosine signaling [58]. In addition, it has been showed that T cells from patients with primary or acquired immunodeficiency display a low CD73 expression and activity [59]. These observations support an important role of CD73 in controlling naïve T cell homeostasis. Expression of CD73 is rapidly reduced upon T cell activation and differentiation. In our study of samples from AML patients, CD73-CD8 T cells are largely antigen-experienced cells and T_EMRA_ express the lowest CD73. Together with our data that CD73-CD8 T cells are less functional, these results indicate an exhaustion pattern of CD73-CD8 T cells.

The results of our study have immense clinical implications. Systemic treatment using blockade antibodies against CD73 is a main approach in the investigational trials targeting CD73-adenosine for cancer control. It is anticipated that inhibition of CD73 on the tumor cells can effectively prevent the release of adenosine, thus enhance anti-tumor immune response and improve clinical outcome. However, our findings that CD73^−^ CD8 T cells express higher frequency of negative receptors such as PD-1, LAG-3, and TIGIT indicate that blocking CD73 may upregulate the inhibitory pathways on CD8 T cells. This could form a potential mechanism of resistance to the CD73-targeting therapies. Our finding is consistent with the report by Tóth et al. that low expression of CD73 on CD8 T cells associates with upregulation of PD-1 and T cell exhaustion in HIV [60]. These observations argue strongly for the combinational treatment by adding immune checkpoint inhibitors to the CD73-targeting approaches.

## Conclusions

In summary, we made a novel observation that downregulation of CD73 is associated with T cell exhaustion. CD73^−^ CD8 T cells display high expression of inhibitory receptors such as PD-1 and TIGIT, increased intracellular expression of Eomes, reduced capacity of cytokine production, and increased susceptibility to apoptosis. Our data highlight the potential of CD73 as a double-edged sword in anti-leukemia immunity and suggest that combination of CD73-targeting agents and other immune checkpoint inhibitors may represent a promising strategy for optimal leukemia control.

## Abbreviations

ADP: Adenosine diphosphate
AML: Acute myeloid leukemia
LAG-3: Lymphocyte-activation gene 3
PBMCs: Peripheral blood mononuclear cells
PD-1: Programmed cell death protein 1
TIGIT: T cell immunoglobulin and ITIM domain
